# Characterization of Properties, In Vitro and In Vivo Evaluation of Calcium Phosphate/Amino Acid Cements for Treatment of Osteochondral Defects

**DOI:** 10.3390/ma14020436

**Published:** 2021-01-17

**Authors:** Lubomir Medvecky, Maria Giretova, Radoslava Stulajterova, Jan Danko, Katarina Vdoviakova, Lenka Kresakova, Zdenek Zert, Eva Petrovova, Katarina Holovska, Maros Varga, Lenka Luptakova, Tibor Sopcak

**Affiliations:** 1 Division of Functional and Hybrid Systems, Institute of Materials Research of SAS, Watsonova 47, 040 01 Kosice, Slovakia; mgiretova@saske.sk (M.G.); rstulajterova@saske.sk (R.S.); tsopcak@saske.sk (T.S.); 2 Department of Morphological Disciplines, University of Veterinary Medicine and Pharmacy in Kosice, Komenskeho 73, 041 81 Kosice, Slovakia; jan.danko@uvlf.sk (J.D.); katarina.vdoviakova@uvlf.sk (K.V.); lenka.kresakova@uvlf.sk (L.K.); eva.petrovova@uvlf.sk (E.P.); katarina.holovska@uvlf.sk (K.H.); 3 Clinic of Horses, University of Veterinary Medicine and Pharmacy in Kosice, Komenskeho 73, 041 81 Kosice, Slovakia; zdenek.zert@uvlf.sk; 4 Hospital AGEL Kosice-Saca, Lucna 57, 040 15 Kosice-Saca, Slovakia; varga99@mac.com; 5 Department of Biology and Physiology, University of Veterinary Medicine and Pharmacy in Kosice, Komenskeho 73, 041 81 Kosice, Slovakia; lenka.luptakova@uvlf.sk

**Keywords:** calcium phosphate cement, amino acid, osteochondral defect, hyaline cartilage, pig model

## Abstract

Novel calcium phosphate cements containing a mixture of four amino acids, glycine, proline, hydroxyproline and either lysine or arginine (CAL, CAK) were characterized and used for treatment of artificial osteochondral defects in knee. It was hypothesized that an enhanced concentration of extracellular collagen amino acids (in complex mixture), in connection with bone cement in defect sites, would support the healing of osteochondral defects with successful formation of hyaline cartilage and subchondral bone. Calcium phosphate cement mixtures were prepared by in situ reaction in a planetary ball mill at aseptic conditions and characterized. It was verified that about 30–60% of amino acids remained adsorbed on hydroxyapatite particles in cements and the addition of amino acids caused around 60% reduction in compressive strength and refinement of hydroxyapatite particles in their microstructure. The significant over-expression of osteogenic genes after the culture of osteoblasts was demonstrated in the cement extracts containing lysine and compared with other cements. The cement pastes were inserted into artificial osteochondral defects in the medial femoral condyle of pigs and, after 3 months post-surgery, tissues were analyzed macroscopically, histologically, immunohistochemically using MRI and X-ray methods. Analysis clearly showed the excellent healing process of artificial osteochondral defects in pigs after treatment with CAL and CAK cements without any inflammation, as well as formation of subchondral bone and hyaline cartilage morphologically and structurally identical to the original tissues. Good integration of the hyaline neocartilage with the surrounding tissue, as well as perfect interconnection between the neocartilage and new subchondral bone tissue, was demonstrated. Tissues were stable after 12 months’ healing.

## 1. Introduction

Degeneration and damage to cartilage, or even subchondral bone, due to primary (mainly genetic, in the absence of a predisposing trauma or disease), as well as secondary, factors (e.g., metabolic, inflammatory, post-traumatic) are in fact a significant social problem; e.g., osteoarthritis (OA) affects about 40 million people in Europe and about 10–13% of adults 60 years of age or older in the US [1,2]. The biomechanical and biochemical relationships between articular cartilage and subchondral bone play an important role in the development of osteoarthritis. In addition, the nerve endings terminate in the subchondral bone, which supports the hypothesis that this part is associated with joint pain [3]. In medical practice, several surgical procedures are applied to treat articular cartilage and subchondral defects, depending on the degree of their damage [4,5,6]. All the methods used have specific drawbacks, which include, e.g., tissue formation with different properties than the original tissue, the need to open another operating field for harvesting of healthy tissue with its possible death at the donor site, the need to cultivate specific cells (e.g., chondrocytes), the possibility of transmitting various diseases between donor and patient, immunogenic response, etc. The hierarchical structure of hyaline cartilage [3,7] significantly complicates the selection of suitable biomaterials and especially their preparation. Due to the low number of active chondrocytes in the cartilage, direct seeding of autologously harvested mesenchymal stem cells or chondrocytes, on developed polymeric porous scaffolds, complicates and makes a more expensive surgical procedure [8,9,10,11,12]. Moreover, the problem of the widespread use of biopolymers for the treatment of osteochondral defects is the insufficient coverage of both the mechanical properties and the characteristic zonal structure of hyaline cartilage.

A progressive solution to problems associated with the lower osteoinductive potential of biopolymers in the subchondral region of the defect may be the simultaneous stimulation of hyaline cartilage and subchondral bone formation through bilayered or multistructural biomaterial arrangement. In addition, substrates may contain growth factors and cytokines to support proliferation and proper differentiation of cells [13,14,15,16,17,18,19,20,21,22,23,24,25,26]. Calcium phosphate cement (CPC) was a reliable subchondral replacement material in rabbit medial femoral condyle defect adjacent to the articular cartilage [27]. α-tricalcium phosphate (αTCP)-based CPC was supplemented with lysine (LYS, up to 2 wt.%) to study its effect on promoting the osteogenic capability [28]. The positive effect of arginine (ARG) on the stimulation of osteoblasts to bone tissue formation was also demonstrated [29]. ARG is a natural inhibitor of cathepsin, which is protease that breaks down cartilage. ARG was significantly depleted in refractory knee OA patients [30]. Enhanced production of collagen II by chondrocytes was verified in culture medium supplemented with lysine, proline and especially glycine [31], as well, the positive effect of ARG and LYS on activity of osteogenic bone osteoblasts was revealed [32]. Hydroxyproline (HYP) is a major substrate for endogenous synthesis of glycine and this pathway is preferred by about 60% higher yield of ATP than from other substrates (e.g., serine, threonine). In collagen, HYP is produced via hydroxylation of proline (PRO) in pro-collagen fibers [33]. On the other hand, PRO, as one of the basic amino acids in collagen, is synthesized in mammals from arginine via arginase I or II [34] or the glutamine pathway [35]. Glycine (GLY) had a strong effect on the expression of the calcified chondrocyte osteopontin marker with calcification of chondrocytes, while no similar effect on osteoblasts was found [36]. Note that large amounts of GLY and PRO are needed for collagen production in cells due to their turnover in collagen synthesis.

In the case of CPC with amino acid additives, a positive influence on osteo- and chondrogenesis can be expected, but few articles [26,27,28] have focused on the treatment and analysis of the healing of osteochondral defects with such CPC composites. The aim of this work was to prepare a one-step synthesis of the tetracalcium phosphate/monetite bone biocements modified with the addition of two different amino acid mixtures, containing the main amino acids of collagen, consequently, and to analyze the influence of adding the amino acid mixture on properties of biocements, as well as to study the effect of biocement on the treatment of artificial osteochondral defects in knees of pigs. It was hypothesized that an enhanced concentration of extracellular collagen amino acids (in complex mixture) in connection with bone cement in the defect site would support the healing of osteochondral defects with successful formation of hyaline cartilage and subchondral bone.

## 2. Materials and Methods

### 2.1. Preparation of Cement Mixtures and Cement Samples

The used preparation procedure allowed us to synthesize the composite biocements with the addition of up to 4 wt.% of amino acids in one step. The selected amino acid mixture in cement contained three basic collagen components (GLY, PRO and HYP) and also ARG or LYS. Tetracalcium phosphate (Ca_4_(PO_4_)_2_O, TTCP) was prepared by annealing of an equimolar mixture of calcium carbonate (CaCO_3_, analytical grade, Sigma-Aldrich, Saint Louis, MO, USA) and dicalcium phosphate anhydrous (DCPA) (CaHPO_4_ (Ph.Eur.), Fluka), at 1450 °C for 5 h. Following this, the TTCP was milled in a planetary ball mill (Fritsch, 730 rpm, agate balls and vessel, d50 = 7 µm) for 2 h. The tetracalcium/monetite powder cement mixture, which was free of amino acids (C cement), was synthesized by an in situ reaction of TTCP and orthophosphoric acid (86% analytical grade, Merck, Darmstadt, Germany) in 80 *v/v* % ethanol (reaction solution) using a planetary ball mill with agate balls and vessel for 30 min [37]. The orthophosphoric acid was added in such an amount to be the Ca/P mole ratio in cements close to 1.67. Two different amino acid/cement powder mixtures—glycine:hydroxyproline:proline:arginine (CAK, mass ratio = 4:2:2:1) and glycine:hydroxyproline:proline:lysine (CAL, mass ratio = 4:2:2:1)—were tested. The amino acids were dissolved in reaction solution with the formation of a saturated solution of phosphate–amino acids. To maintain sterile conditions for in vivo testing, amino acid solutions were filtered through a 0.2 μm membrane filter (Millipore, PVDF, Darmstadt, Germany) in a laminar box (ESCO, class II, Esco Micro Pte Ltd, Singapore, Singapore); the sterile solution was then added to TTCP (sterilized at 160 °C/2 h) and the suspension was milled under the same conditions, as in the case of C cement, and dried at 100 °C under sterile conditions. Resulting CAK and CAL cement powder mixtures contained 4 wt.% of amino acids (glycine, L-proline, 4-hydroxy-L-proline, L-lysine or L-arginine (all Sigma)). The cements were prepared by the mixing of powder mixtures with 2% NaH_2_PO_4_ (as hardening liquid, sterile solution) at P/L ratio = 2.

The amount of released calcium and phosphorus, as a composition of hardened cements (Ca/P ratio), was determined by ICP (Horiba Activa, HORIBA Jobin Yvon Inc., Park Avenue, Edison, NJ, USA) after their dissolution in HNO_3_ (20%, analytical grade, Merck, Darmstadt, Germany).

### 2.2. XRD Phase Analysis, Setting Time and Microstructure of Cements

The phase composition of samples was analyzed by X-ray diffraction analysis (Philips X’ PertPro, Malvern Panalytical B.V., Eindhoven, Netherlands, using Cu Kα radiation, 40 kV, 50 mA, 2θ range 10°–60°) and FTIR spectroscopy (Shimadzu, IRAffinity1,Kyoto, Japan, 400 mg KBr + 1 mg sample). The content of starting TTCP phase in hardened cements was determined by semiquantitative analysis [38].

The microstructure of cement surfaces was observed by field emission scanning electron microscopy (JEOL, FE SEM JSM-7000F, Tokyo, Japan). 

### 2.3. Soaking Cements—pH Measurement; Release of Amino Acids and Ca; Phosphate Ions from Cements

The changes of pH during the cement soaking at various times (4, 24, 96, 120 and 168 h) were measured using 500 mg CPC pellets (6 mm D × 12 mm H, prepared by the molding of cement pastes in stainless steel mold) hardened for 10 min in 100% humidity and following immersion in 50 mL simulated body fluid (SBF) [39] solutions at 37 °C. The pH solution was measured by a pH-meter (Xylem-WTW, Inolab 720, Weilheim, Germany) with the SenTix41 combined electrode. The ionic and amino acid release was carried out by soaking of cement pellets in 0.9% NaCl solution at 37 °C (500 mg of cement/15 mL of solution). The total concentrations of released calcium and phosphorus to solution were analyzed using ICP (Horiba Activa, HORIBA Jobin Yvon Inc., Park Avenue, Edison, NJ, USA) after 4, 24, 96, 120 and 168 h of soaking. The physiological solution has approximately the same ionic strength as SBF but does not contain other ionic species that could be affect the HPLC analysis, including the damage of the chromatographic column due to precipitation of calcium phosphates. Moreover, very small differences in Ca or P concentrations during soaking may not be observed in the SBF solution, which is saturated in relation to hydroxyapatite. 

The release of amino acids was measured by a HPLC ionex chromatography (Watrex with Clarity station, column Nucleosil SA 100-5, Macherey-Nagel, Germany) at isocratic conditions with flow rate of the mobile phase (0.15 M hydrogen diammonium phosphate solution, pH = 3.7) equal to 1 mL/min using a UVVIS detector (SYCAM, Eresing, Germany) at 210 nm wavelength.

### 2.4. Measurement of Compressive Strength, XRD Phase Analysis, Setting Time and Microstructure of Cements

The cement pastes were molded into pellet form (6 mm D × 12 mm H) for measurement of compressive strength. The samples were soaked in SBF solution at pH = 7.4 and 37 °C for 7 days after setting in 100% humidity at 37 °C for 10 min, which is sufficient time for samples to not be disintegrated after immersion into solution. The compressive strength (mean of 5 samples) of dry (after drying samples at 100 °C) and wet (immediately after soaking) samples was measured on a universal testing machine (5 kN load cell, LR5K Plus, Lloyd Instruments Ltd. West Sussex, UK) at a crosshead speed of 1 mm/min.

The final setting times of the cement pastes were evaluated using the tip (1 mm diameter) of a Vicat needle with a 400 g load (according to ISO standard 1566 [40]); it failed to make a perceptible circular indentation on the surface of the cement. The relative density of samples was calculated from the measured dimensions and weight of samples. The theoretical density of hydroxyapatite (3.15 g/cm^3^) was used for calculation. 

### 2.5. Preparation of Cell Extracts and In Vitro Cytotoxicity Testing of Extracts 

The cement extracts were obtained by soaking of cement pastes in the complete osteogenic differentiation culture medium composed of the α-modification Eagle’s minimum essential medium (EMEM, Biosera, 10% FBS, osteogenic supplements L-ascorbic acid 50 µg/mL, 50 nM dexamethasone, 10 mM β-glycerophosphate and 1% penicillin, streptomycin, amphotericin (all Sigma-Aldrich, Saint Louis, MO, USA) in an incubator at 37 °C for 24 h using a sterile 50 mL polypropylene centrifuge tube. A ratio of 0.2 g cement powder per mL of medium (A) (in accordance with ISO 10993-12:2012 [41]) or 0.1 g cement/mL medium (B) (in the case of long-term testing of up to 15 days) was used. Extracts were sterilized by filtration through a 0.2 µm membrane (Millipore, PVDF).

MC3T3E1 cells were harvested from culture flasks by enzymatic digestion and resuspended in culture medium. The cell suspension was adjusted at a density of 1.0 × 10^5^ cells/mL. Briefly, 1.0 × 10^4^ of pre-osteoblastic MC3T3E1 Subclone 4 cells (ATCC CRL-2593, Manassas, VA, USA) cells were suspended in 100 µL of EMEM (Biosera) + 10% FBS, 1% antibiotic solution and seeded into each well of the 96-well cell Grade Brand microplate (adherent wells) and cultured to a semi-confluent monolayer at 37 °C, 95% humidity, and 5% CO_2_ in an incubator for 24 h. Subsequently, the culture medium in the wells was replaced with 100 µL of 100% extract (A,B). All experiments were carried out in triplicate, and the cells in the wells with extract-free complete culture medium were considered as a negative control. After 24 h culturing, the A extracts were replaced with fresh culture medium and the in vitro cytotoxicity was evaluated (ISO 10993-5:2009 [42]) by the MTS proliferation test assay (cell titer 96 aqueous one solution cell proliferation assay, Promega Madison, WI, USA). The absorbance of formazan was determined by a UV–VIS spectrophotometer (Shimadzu, Kyoto, Japan,). The B extracts were used for the long-term osteoblast viability testing where culture extracts were exchanged every two days. The distribution and morphology of the osteoblasts on the surface of hardened CAK and CAL cements after culture for 9 days in osteogenic medium were visualized with live/dead staining (fluorescein diacetate/propidium iodide) by an inverted optical fluorescent microscope (Leica DM IL LED, blue filter, Heerbrugg, Switzerland) and by SEM after dehydration of samples in ethanol and coating with carbon.

### 2.6. Analysis of ALP Activity of Osteoblasts in Extracts and Gene Expression

The ALP activity of osteoblasts was determined in cell lysates after lysis in solution containing 0.1% Triton X-100, 1 mM MgCl_2_, 20 mM Tris. The cell lysates were transferred into 1.5 mL microcentrifuge polypropylene tubes, frozen at −20 °C and centrifuged at 10,000 RPM for 10 min after thawing. The 100 µL of cell supernatant was added to 100 µL of p-nitrophenyl phosphate in diethanolamine buffer (0.5 mM MgCl_2_, pH 9.8) and incubated at 37 °C. The reaction was stopped after 60 min with 50 µL of 3 M NaOH. The amount of p-nitrophenol produced during the ALP enzyme catalysis of the p-nitrophenyl phosphate substrate was determined from the calibration curve of p-nitrophenol at 405 nm using the UV–VIS spectrophotometer. The ALP activities were expressed in nanomoles of the p-nitrophenol produced per 1 min per µg of proteins. The content of proteins in lysates was evaluated by Bradford´s method with Coomasie blue G250 as the complexing agent [43]. The statistical evaluation of results (n = 4) was performed using two-way ANOVA analysis at level α = 0.05. 

For the extraction of total RNA, approximately 1 × 10^6^ cells were used. Total RNA from each cell culture was extracted using the RNeasy Mini Kit (Qiagen, Germantown, MD, USA) following the manufacturer’s instructions. Contaminating genomic DNA was digested using the RNase-free DNase set (Qiagen, Germantown, MD, USA). The RNA quality and yields were analyzed using the Nanodrop spectrophotometer (Thermo Scientific, Germantown, MD, USA). Complementary DNA (cDNA) synthesis was performed using protocol for RT2 First Strand Kit (Qiagen, Germantown, MD, USA), where 1 µg of total RNA was used (after the genomic DNA elimination step) to prepare 20 µL of cDNA. cDNA was then used for real-time PCR experiments. The quantification of genes of interest in the cDNA samples was performed using primers for osteopontin (OP), osteonectin (ON), collagen I and ALP (Table 1).

Twenty-five microliter reaction mixtures, each consisting of triplicate samples of cDNA, specific primer mix and RT2 SYBR Green qPCR mastermix (Qiagen, Germantown, MD, USA), were setup in each well of a 96-well reaction plate (Roche, Basel, Switzerland). cDNA for β-actin was used as endogenous control for calculating fold differences in the RNA levels of cells treated with CAK or CAL vs. C cement extract by the 2−ΔΔCT method. The plate was sealed using an optical adhesive cover (Roche, Basel, Switzerland) and was placed in the LightCycler 480 II real-time PCR system machine (Roche, Basel, Switzerland). The real-time PCR was performed under the following conditions: initial incubation at 95 °C for 10 min, amplification in 45 cycles at 95 °C for 15 s, followed by 60 °C for 1 min. The amplification specificity was checked by the generation of a melting curve. 

### 2.7. Chorioallantoic Membrane Model for Angiogenesis

Currently, avian embryos are used in biomaterial research because the chorioallantoic membrane (CAM) of the avian embryo provides a simple and effective alternative model for assessing the biocompatibility of new biomaterials. The CAM is highly vascularized, constituting both mature vessels and capillaries, and is easily accessible for orthotopic implantation of biomaterials without initiating an immune reaction from the developing embryo [45]. This alternative animal model represents an intermediate step for testing biomaterials between the simple model (in vitro) and the complex in vivo system (rodents, large animals) in accordance with the principles of the 3Rs. Furthermore, the CAM model is exempt from the horizontal legislation of the protection of animals used for scientific purposes (2010/63/EU), as well as the applicable laws in the United States [46].

Fertilized Japanese quail (Coturnix japonica) eggs (N = 300) were purchased from an animal farm (Kosice, Slovakia) and delivered via courier in a temperature-controlled manner to ensure egg viability and quality. Fertilized eggs were incubated in a forced draught incubator at 38.2 °C and 50–60% relative humidity. On embryonic day (ED) 3, the surface of the eggs was gently cleaned with 70% ethanol in a UV-cleaner box. The eggs were opened, and the embryos were transferred into six-well tissue culture plates (TPP, Trasadingen, Switzerland) [47] and returned to the humidified incubator for the next 3 days. On ED6, dead embryos were discarded and biocements, C, CAK, and CAL (1 piece per embryo; 2 mm × 2 mm, prepared from sterile cement pastes and hardened at 37 °C in 100% humidity), were gently put on the CAM surface of the survived embryos. Following biomaterial implantation, the embryos were incubated further at 38.2 °C for the next 72 h. On ED9, the vascularization of CAM around biomaterials was evaluated. The vascular index was measured as a difference between the number of vessels at the beginning of treatment and the number of vessels 72 h after implantation [48]. The CAM blood vessel formation was observed using a stereomicroscope Olympus SZ61 and digital camera ARTCAM-300MI (Olympus, Tokyo, Japan). The implantation of biomaterials was repeated three times. 

Results were expressed as mean ± SD (standard deviation). Statistical analyses of data were performed using a paired T-test and one-way ANOVA, followed by Tukey’s test. Values of *p* < 0.05 were considered statistically significant.

### 2.8. Surgical Procedure, Post-Operative Care and Rehabilitation

The experiment was conducted in accordance with the institutional guidelines of the State Veterinary and Food Administration of the Slovak Republic (study No. 4650/17-221). Six female large white pigs (breeding farm PD Agro, Michalovce, Slovakia), 5 months old with a mean weight of 50–60 kg, were used. Animals were residing in the Clinic of Swine of the University of Veterinary Medicine and Pharmacy in Kosice (Kosice, Slovakia). All pigs had free access to food and water during the in vivo testing. 

Sedation of the animals was conducted with a mixture of atropine 0.02 mg/kg body weight (Atropine sulfate 1 mg/mL solution for injection, FATRO S.p.A, Bologna, Italy), azaperone 5 mg/kg body weight (Stresnil 40 mg/mL, Janssen Pharmaceutica, Beerse, Belgium) and buthorphanol 0.02 mg/kg body weight (Butomidor 10 mg/mL, Richter Pharma, Wels, Austria) administered intramuscularly. This was later followed by thiopental 15 mg/kg body weight (Thiopental VUAB 1.0 g, VUAB Pharma a.s., Roztoky, Czech Republic), administered intravenously for induction of general anesthesia. After aseptic preparation and draping of the skin, a 12 cm long incision was made on the porcine left stifle joint, from the medial patellar ligament to the tibial tuberosity, distally. This surgical wound was used for access to the medial femoral condyle where a defect (diameter of 10 mm, depth of 10 mm) was created in the articular cartilage and subchondral bone by the osteochondral autograft transfer system (OATS, Arthrex, Naples, FL, USA). Into the drill hole was then inserted the sterile biocement pastes (C, CAK or CAL, two animals for each cement). The muscles and skin were closed in layers with sutures immediately after the defect filling and covered by a liquid aluminum spray bandage. The X-ray detection was used for confirmation of the completely defect filling. Animals received an intramuscular injection of broad-spectrum antibiotics oxytetracycline dihydrate 1 mL/10 kg body weight (Alamycin LA a.u.v., Norbrook, Newry, UK) postoperatively once every second day for 7 days and an intramuscular injection of non-steroidal anti-inflammatory drug flunixin meglumine 2.2 mg/kg body weight (Flunixin a.u.v., Norbrook, Newry, UK) postoperatively once a day for 7 days. After surgery, the pigs were housed individually, and they were allowed to recover in a solid pen with straw bedding. Animals were euthanized after 3 months post-surgery. 

### 2.9. Macroscopic Evaluation, Histological and Radiographic Analysis

The regenerated tissue from the artificial defect site was obtained by collecting a 10 mm diameter and 10 mm long bone cylinder using the osteochondral autograft transfer system (OATS, Arthrex, Naples, FL, USA), fixed in neutral formalin per one week and soaked in chelaton for demineralization per one month. In the next step, samples were dehydrated in 70–100% ethanol-graded series, paraffin–embedded, sectioned at 7 µm thickness with microtome and prepared for staining with hematoxylin and eosin (H&E), Alcian blue and Picrosirius red. Tissue sections were stained by H&E for visualization of cells, cell nuclei, cartilage matrix and tidemark; Alcian blue detected proteoglycans (glycosaminoglycans); Picrosirius red visualized the collagen fibers in histological sections. 

Collagen II was identified immunohistochemically using a primary antibody Rabbit polyclonal anti collagen antibody (Abcam) and a secondary antibody in DB DET SYS kit (Biotech) according to the manufacturer’s instructions. Visualization of collagen II was carried out with DAB (3,3’-diaminobenzidine) (DAKO) substrate. All histological samples were evaluated using a light microscope (Olympus CX 23).

The investigated parts of the bone (distal epiphysis of the left porcine femur, medial condyle) were harvested after 3 months post-surgical procedure to assess the healing process via the analysis of neocartilage and new subchondral bone tissue by the magnetic resonance imaging (MRI) and X-ray analysis. The scanning protocol involved slices in ultra-high resolution with a thickness of 3.5 mm for MRI (1.2 T Hitachi Oasis, Open system, Hitachi Medical Systems Holding AG, Tokyo Japan) and X-ray (Philips Digital Diagnost, Delft, The Netherlands) radiographs.

## 3. Results

### 3.1. XRD and FTIR Analysis

**Figure 1:**
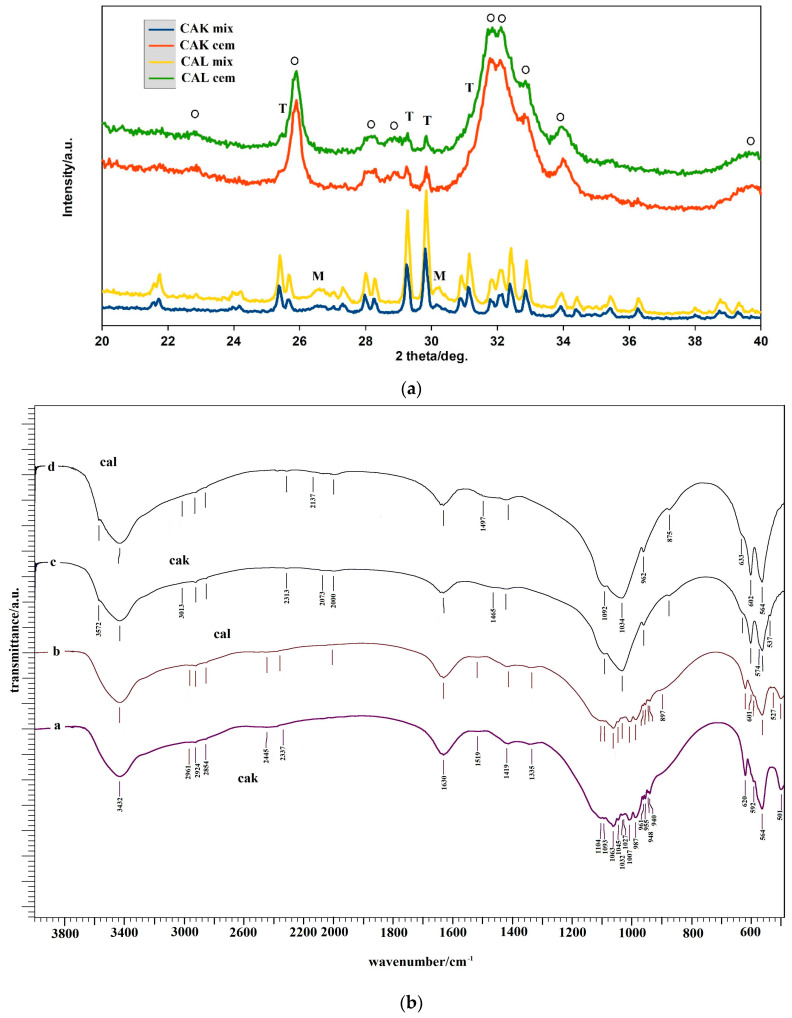
XRD patterns (**a**) (M-monetite, ○-HAP, T-TTCP) and FTIR spectra (**b**) of cement mixtures and cements after 7 days soaking at 37 °C in SBF (a–CAK mix, b–CAL mix, c–CAK cem, d–CAL cem).

In Figure 1A, the XRD patterns of the origin cement powder mixtures and CAK, CAL cements after 7 days soaking at 37 °C in SBF are shown. The XRD analysis verified the presence of pure tetracalcium phosphate phase (JCPDS25-1137) and the formation of fine monetite phase (with reflections from (020), (−220) and (−112) monetite planes (JCPDS09-0080) at 2θ equal 26.51° and 30.21°) during reaction milling. On the other hand, from the comparison of XRD patterns of CAK and CAL cements, the results show that the final product of the transformation of cements was calcium deficient nanocrystalline hydroxyapatite (PDF4 01-071-5048) with a small carbonate substitution (HAP). In both cements, approximately 8% of starting TTCP phase was found after setting in SBF for 7 days. The formation of HAP also verified the chemical analysis of cements after 7 days setting in SBF, where the Ca/P ratio was close to 1.65 ± 0.02. Note that no other phases were identified in patterns.

FTIR spectra of cement mixtures and cements are compared in Figure 1B. In the FTIR spectra of the starting cement mixtures, the ν_3_ and ν_1_ stretching vibrations of the ${PO}_{4}^{3-}$ group at 1062, 1046, 1007 and 987 cm^−1^; ν_4_ and ν_2_ deformation O–P–O vibrations of origin TTCP [49]; shoulders at 1130 and 900 cm^−1^, characteristic for ν_3_ stretching vibrations of P–O and P–O(H) monetite bonds, are found [50]. The peaks from OH plane bending monetite vibrations at 1408 and 1347 cm^−1^ overlap the stretching vibrations ν_sym_(COO^−^) and wagging deformation vibrations ω(CH_2_) of glycine at 1413 and 1336 cm^–1^, respectively. In the spectra of the starting CAK and CAL cement mixtures, the peaks assigned to glycine can be clearly visible because this amino acid represents about 50 wt.% from total amino acid content, but its molar fraction is much higher due to a lower molecular mass compared to the others. From the analysis of the spectra, the results show that the glycine bands were closed to their zwitterionic form [51], but shift due to higher frequencies of ν_assym_(COO^−^) vibrations from 1615 to 1630 cm^−1^, which were measured after adsorption on the calcium phosphate particles. No changes in the locations of other characteristic bands from ν_sym_(COO^−^) at 1413 cm^−1^; ω(CH_2_) at 1336 cm^−1^; δ_s_(NH_3_^+^) at 1515 cm^−1^, were identified. The same effect was observed after the interaction of glycine in zwitterionic form with brushite, which corresponded with the interaction of glycine with calcium [52].

The large change can be visible in FTIR spectra of hardened cement samples, which demonstrates almost full transformation of original calcium phosphates to hydroxyapatite. Thus, the characteristic vibrations of the ${PO}_{4}^{3-}$ group in hydroxyapatite arise from antisymmetric (ν_3_) and symmetric (ν_1_) P–O stretching vibrations at 1032, 1091 and 962 cm^−1^; O–P–O bending (ν_4_) vibrations at 565 and 601 cm^−1^ were revealed in spectra. In addition, the weak distinguished liberational mode of the OH group at around 630 cm^−1^ and a low intense peak from stretching vibrations of the OH group in hydroxyapatite at around 3570 cm^−1^, as well as peaks corresponding to ν_2_ and ν_3_ vibrations of the ${CO}_{3}^{2-}$ group at 1480–1460, 1420 and 875 cm^−1^ characteristic for the carbonated hydroxyapatite type B-type with ${CO}_{3}^{2-}$ substitution for ${PO}_{4}^{3-}$ groups [53,54], were observed in spectra. Note that bands from the vibrations of amino acids in hardened cements were strongly reduced after 7 days soaking in SBF.

### 3.2. Microstructure of Cements

**Figure 2:**
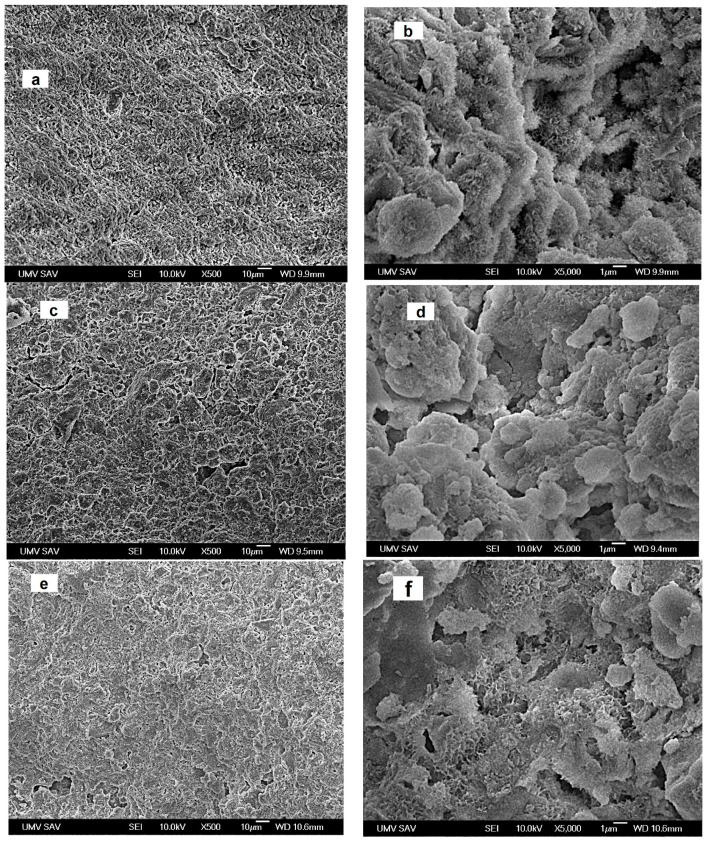
Microstructures of cements after 7 days setting in SBF ((**a**,**b**)–C; (**c**,**d**)–CAK; (**e**,**f**)–CAL).

Microstructures of cements after 7 days setting in SBF are shown in Figure 2. A low number of larger 10–15 µm pores and a high fraction of irregularly shaped micropores of 1–3 µm size can be found in the microstructure of C cement. The plate- or needle-like nanohydroxyapatite particles with a 100–200 nm length formed globular (about 1 µm) agglomerates mutually interconnected via the network of boundary surface particles (Figure 2a,b). In the macrograph of CAK cement in Figure 2c,d, a few of the bigger irregularly shaped macropores (10–20 µm size) and spherical agglomerates with diameters up to 20 µm were revealed. Simultaneously, very fine nanohydroxyapatite particles of the spherical shape, with dimensions not exceeding 100 nm and joined to compact agglomerates, were observed in more detailed images. Note that the needle-like hydroxyapatite nanoparticles practically vanished from the microstructure. A similar fraction of larger micropores and a high portion of 1–3 µm micropores, as in CAK, were found in CAL cement. Contrary to observation of the CAK microstructure, the hydroxyapatite agglomerates of 5–10 µm size had a more irregular shape, which can be clearly visible in zoom (Figure 2e,f). In addition, two morphologically different types of agglomerates, consisting of the fine spherical nanoparticles joined to more compact objects and very thin, plate-like particles (thickness less than 50 nm) connected to aggregates with lower density, were identified in CAL microstructure. The relative densities of C, CAK and CAL samples were 48.3 ± 0.3%, 44 ± 0.5% and 43 ± 0.4%, respectively.

### 3.3. Changes in pH during Cement Soaking, Release of Amino Acids from Cements, Setting Time and Compressive Strength

The measurement of the pH changes with the soaking time of the cement samples in SBF clearly demonstrated a more basic character of the C cement as compared with CAK and CAL cements. After 24 h of C cement soaking, the pH of SBF rose to 7.70 and no other change was found in the prolonged soaking period. On the other hand, the values of pH during CAK and CAL soaking were practically identical (around 7.5) and did not change with soaking time, which demonstrates the significant buffering capacity of amino acids.

**Figure 3:**
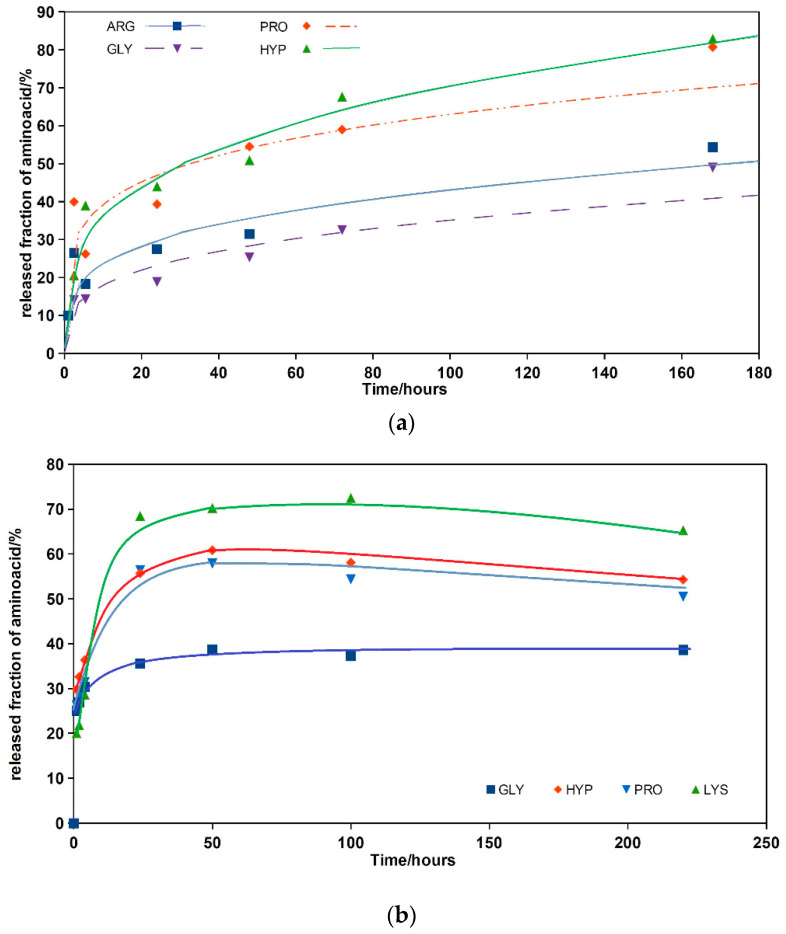

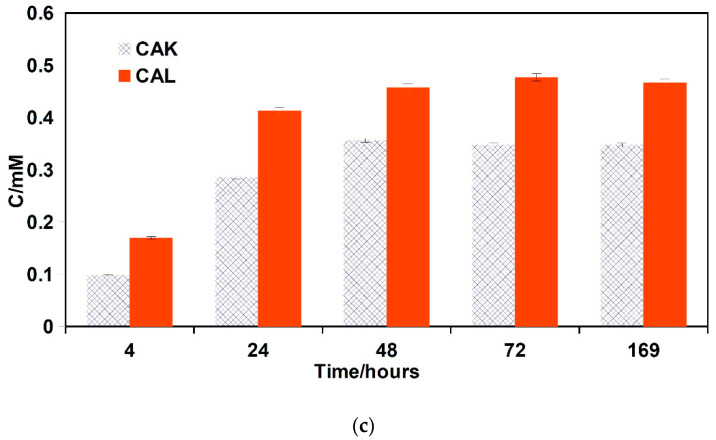
Release of amino acids (CAK—(**a**), CAL—(**b**)) and calcium (**c**) from cements to 0.9% NaCl solution at 37 °C.

Figure 3a,b depict the release kinetics of amino acids from CAK and CAL cements during soaking in SBF at 37 °C. From the comparison of curves, the results show significant differences in the release rate of all amino acids, especially during the first 48 h of soaking the cement pellets. The release of amino acids was very fast up to 10 h of soaking in both cements, which represents the burst effect typical for the release of molecules adsorbed on the outer surfaces of the samples that included micropores. In the CAK cement with a denser and more compact microstructure, an almost linear dependence of amino acid release on time was revealed after 6 h of soaking. The highest-released fractions of amino acids from this cement were the HYP and PRO fractions with around 80% release of the total amount after 168 h contrary to about 50% of the released amount of GLY and ARG. In the case of CAL cement, the plateau on the release curve of all the amino acids was achieved after 50 h from the cement immersion to SBF, with the release of about 40% of GLY, 70% of LYS, 55 and 60% of HYP and PRO, respectively. A further desorption of amino acids was stopped, and the system was in a steady state.

From ICP analysis, the results show that a low concentration of phosphates (<0.1 mM) was only released from both cements contrary to calcium concentration, which gradually rose to approximately 0.35 and 0.45 mM for CAK and CAL cements, respectively, after 48 h soaking (Figure 3c).

The final setting time of the cements was close to 5 ± 1 min regardless of the cement type, and the cements were resistant to disintegration in SBF after 8 min of hardening. The compressive strength (CS) of C, CAK and CAL cements was 40 ± 3, 14 ± 1.2 and 14 ± 0.7 MPa, respectively.

### 3.4. Cytotoxicity of Cement Extracts and ALP Activity of Osteoblasts, Gene Expression

**Figure 4:**
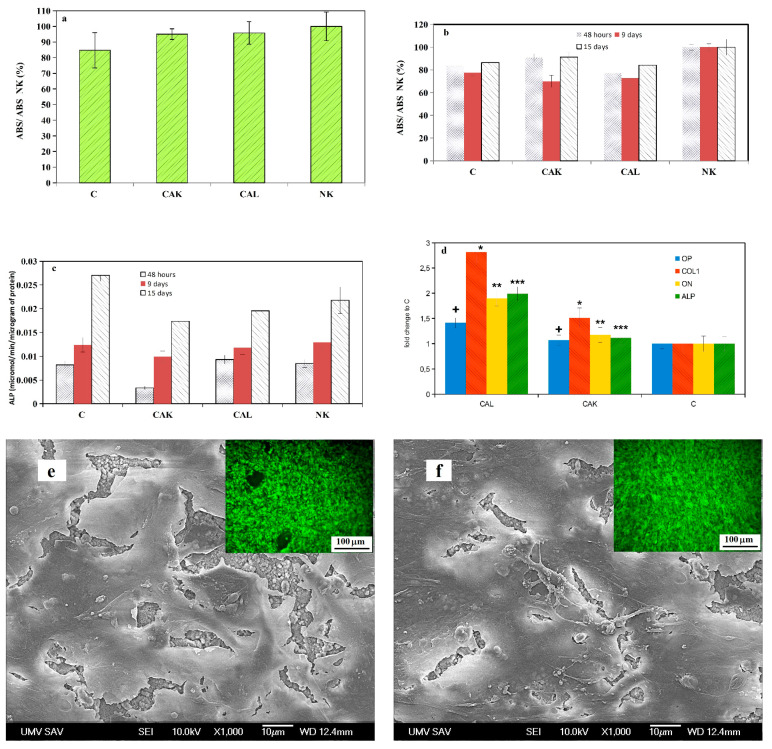
Viability of osteoblasts cultured in A cement extracts for 24 h (**a**), B cement extracts during the prolonged cultivation period of up to 15 days (**b**), ALP activity of osteoblasts cultured in B cement extracts (**c**), relative gene expression of COL1, ON, OP; ALP in osteoblasts cultured for 7 days in B extracts of CAL (statistically significant differences, *p* < 0.05) (**d**); SEM micrographs and live/dead staining of osteoblasts cultured for 9 days in osteogenic medium on CAK (**e**) and CAL (**f**) hardened cements.

The viability of osteoblasts cultured in A cement extracts for 24 h (measured according to ISO 10993-5:2009 [42]) is shown in Figure 4a. From the comparison of the formazan absorbances of samples after MTS testing in relation to the absorbance of negative control (osteoblasts cultured in medium), the results clearly show that the viability of cells achieved a 70% level of viability cells in negative control. Thus, A cement extracts were non-cytotoxic for osteoblasts. For a prolonged period of viability testing of osteoblasts, B cement extracts (50% amount cements used in A extracts) were used because the concentration of individual species released from cements is gradually diluted in body fluids after implantation to the body. No cytotoxicity of B cement extracts was revealed during the prolonged cultivation period of up to 15 days, despite there being lower values of relative formazan absorbance than in the negative control (Figure 4b). The ALP activity of osteoblasts cultured in B cement extracts rose with culture time, but the highest values were measured in C extract (statistically significant differences with all ALP activities from other samples at a given time, *p* < 0.05) (Figure 4c). The statistically significant difference (*p* < 0.01) between ALP activities of osteoblasts in CAK and CAL (0.0033 and 0.0094 µmol/min/µg of proteins in CAK and CAL extracts, respectively) extracts was identified after 48 h of culture only.

The relative gene expression (Figure 4d) clearly demonstrated higher up-regulation of COL1, ON, OP and ALP in osteoblasts cultured for 7 days in B extracts of CAL than in CAK or C cements (statistically significant difference, *p* < 0.01), whereas the statistically significant COL1 over expression (*p* < 0.05) in osteoblasts was only identified in CAK extract as compared to C extract.

The distribution and morphology of osteoblasts on the surface of CAK and CAL cements (Figure 4e,f), after 9 days of cultivation in osteogenic media, revealed a complete coating of cements with a multilayer of live cells which verified the non-cytotoxic character of cements.

### 3.5. In Vivo Angiogenesis 

**Figure 5:**
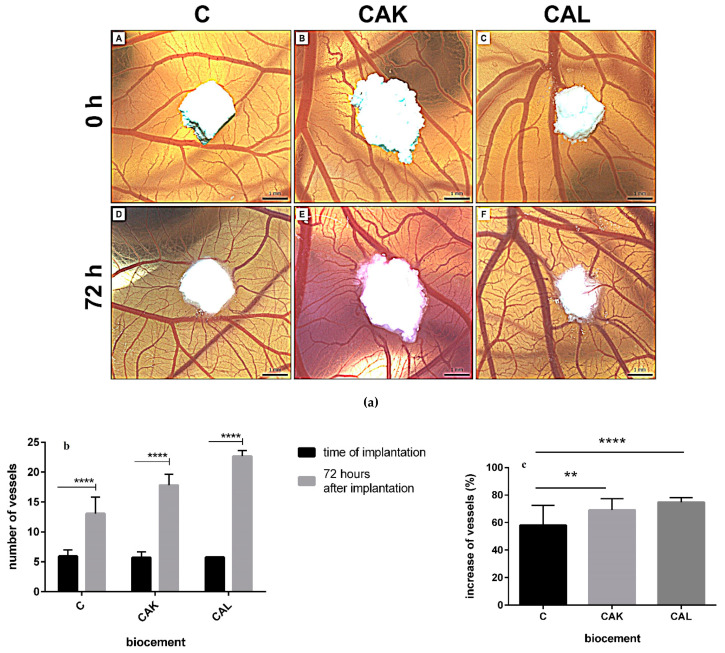
Quail chorioallantoic membrane (CAM) assay (**a**): representative photographs with the growing of vessels toward the biomaterials in the time of implantation (0 h; A–C) and 72 h after implantation (D–F), scale bar = 1 mm; quantification of quail CAM vessels at 0 h and 72 h after implantation of biocements (**b**). Results are summarized in the graph as the vascular index for each tested biomaterial. The graph shows data presented as mean ± standard deviation of three independent experiments (**** *p* < 0.0001 per each biomaterial); angiogenic activity of biocements 72 h after their implantation (^**^ *p* < 0.01; **** *p* < 0.0001) (**c**).

The in vivo angiogenic activity of tested biocements was evaluated using the CAM assay. All tested biocements (C, CAK, CAL) significantly induced growing of vessels toward the biocement 72 h after implantation (*p* < 0.0001; Figure 5a,b). Furthermore, the differences between angiogenic activity of biocements after implantation were verified. In the sample, C biocement increased the number of vessels by 58% after implantation and there was a slight induction of angiogenic activity compared to other CAK and CAL cements with a 69% and 75% increase in the number of vessels after implantation, respectively. Regarding this evaluation, angiogenic activities of CAK and CAL cements were significantly higher than for C cement (CAK-*p* < 0.01; CAL-*p* < 0.0001; Figure 5c).

### 3.6. In Vivo Macroscopic Evaluation

**Figure 6:**
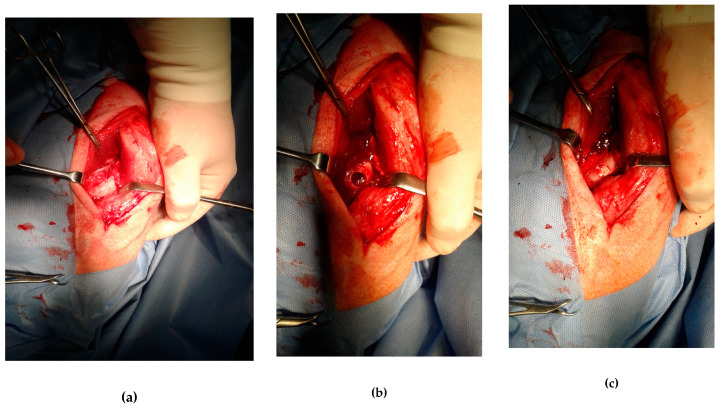

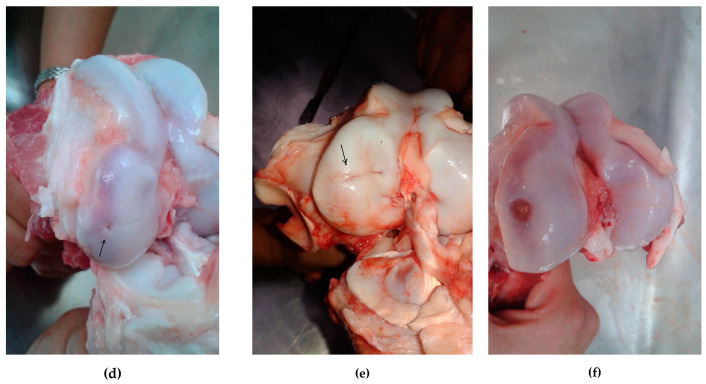
The operating procedure (top). Left porcine stifle joint, medial condyle (**a)**, created defect (Φ 10 mm) in the articular cartilage and subchondral bone (**b**), drill hole was filled by biocement (**c**). Bottom: macroscopic evaluation of the osteochondral defect healing with biocements (**d**) CAL, (**e**) CAK, (**f**) C.

The operating procedure is documented in Figure 6a–c. All animals were moving normally, no gait instabilities due to severe limp were found at any point after the surgery. Any macroscopic signs of the inflammation or deep infection were observed on the treated knee. Regenerated tissue of both the cartilage and subchondral bone in the defect site was observed in all experimental animals. The original defect was visible macroscopically on each examined knee but the difference with native cartilage became increasingly difficult with healing time. The formation of new cartilage began on the margin and progressed directly to the center of the osteochondral defect. In the defect filled by C and CAK cements, the irregularity in cartilage surface occurred compared to smooth cartilage with the uniform surface texture formed in the defect treated with CAL cement (Figure 6d). The sample from defect filled with C biocement revealed the incomplete cartilage defect healing and irregular concave cartilage surface with a small amount of fluid within the center of the defect (Figure 6e). In the defect with CAK cement, a smooth, homogeneous surface of the cartilage, the transverse superficial fissure in the center of the defect, was revealed (Figure 6d). The color of the newly formed cartilage in the samples CAL and CAK was white, contrary to the translucent character of the tissue in the defect treated with C cement. The neocartilage integration with adjacent native cartilage in the defect border was visible after the healing of defects treated with CAL and CAK cements, because in the case of the C sample, the defect depth was only verified to be 50% repaired. The neocartilage did not reach the edge of the native articular cartilage. Despite the macroscopic image of the regenerated cartilage, hyaline cartilage was typical in all of the samples.

### 3.7. Histological Analysis

**Figure 7:**
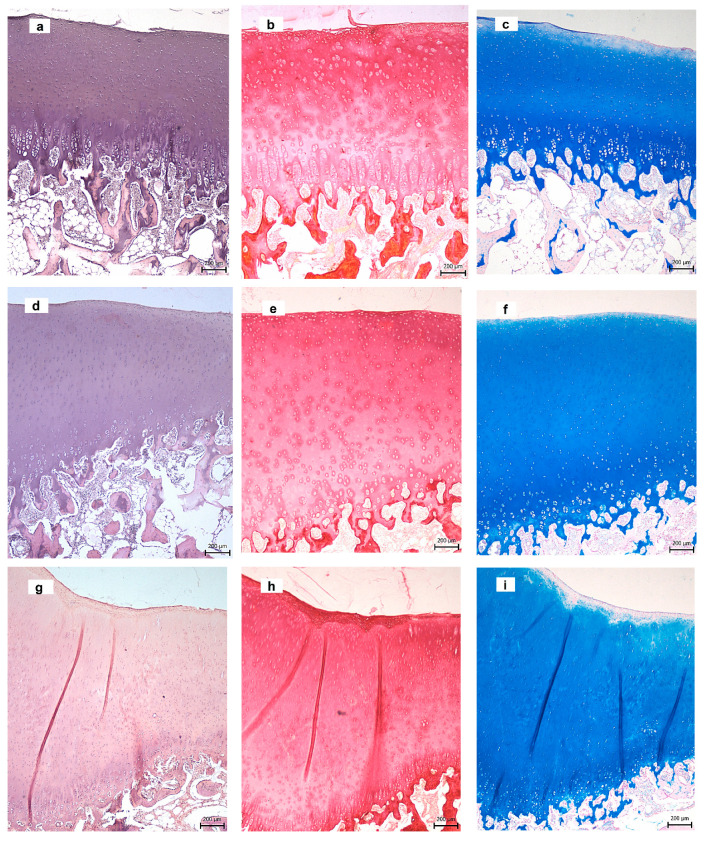

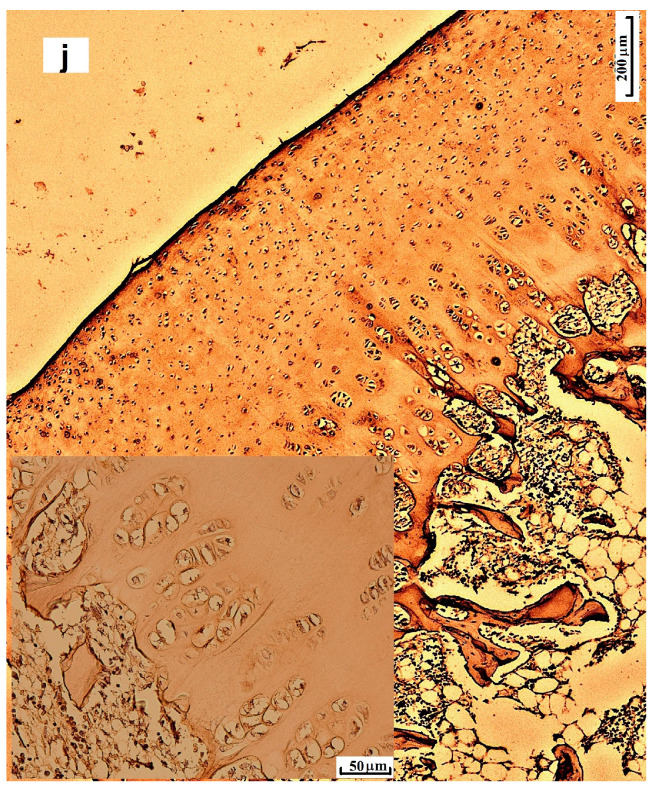
Histological analysis of tissues in defects after 3 months’ healing ((**a–c**) CAL; (**d–f**) CAK; (**g–i**) C) stained with hematoxylin & eosin (**a,d,g**)), Picrosirius red (**b,e,h**), Alcian blue (**c,f,i**), collagen II in CAL (**j**). (Arrows—defect sites.)

In the examined knee with CAL biocement (Figure 7a–c), a defect was completely filled with hyaline cartilage after 3 months of healing. The surface of the neocartilage was slightly irregular with flaking layers containing cells. Chondrocyte morphology and cell density in all layers were similar to the native cartilage. The surface zone consisted of flattened chondrocytes arranged parallel to the cartilage surface. The oval chondrocytes were detected in the transitional zone and randomly distributed within the matrix. The intensity of Alcian blue staining, which indicated a higher content of proteoglycans, increased with depth from superficial towards radial zones (Figure 7c). In Picrosirius red staining (collagen staining), the trend was reversed, which demonstrates a high-intensity staining (collagen content) at the surface and lower intensity towards the calcified layer (Figure 7b). The radial zone contained small round chondrocytes that were arranged into columns, parallel to the long axis of the bone. The calcified zone, composed of a calcified matrix with a low fraction of cells and underlying bone plate, formed a continuous mineralized layer (Figure 7a). The subchondral bone had the characteristic physiological structure.

Similar to the case of CAL cement, the subchondral defect in animals treated with CAK biocement was completely filled with new hyaline cartilage (Figure 7d–f). The articular surface was smooth and intact. The structure of the neocartilage was comparable with the native cartilage and the superficial zone composed of flattened chondrocytes. The staining intensity of Alcian blue was close to the intensity of the physiological sample. The subchondral bone was intact. The distribution of collagen II in cement immunohistochemically stained is shown in Figure 7j; type II collagen was expressed in all cartilage zones in the cross section and was also clearly visible in the pericellular matrix of chondrocytes.

The C biocement was also completely resorbed and replaced by the new tissue similar to native articular cartilage (Figure 7g–i), but the zone structure was disturbed and more heterogeneous. In the superficial zone of the central part of cartilage, a number of chondrocytes decreased, and acellular zones were evident. A large amount of GAGs was found in matrix stained with Alcian blue.

### 3.8. Radiographic Analysis

**Figure 8:**
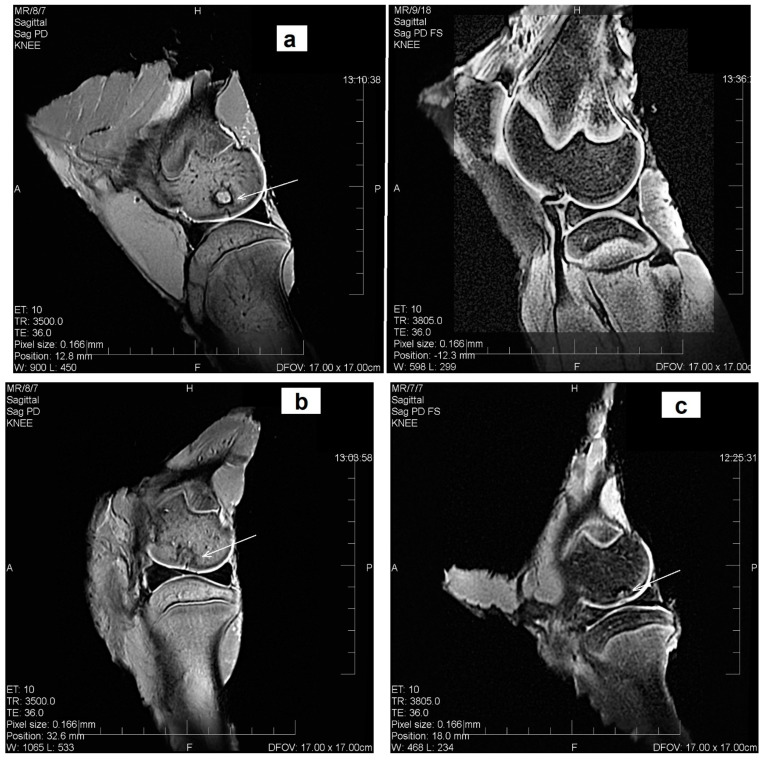

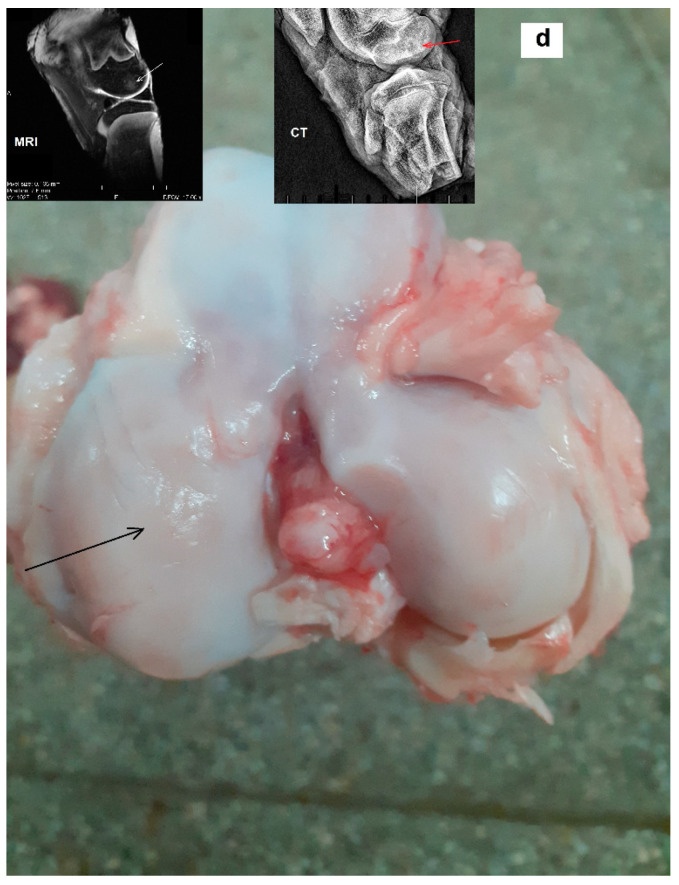
MRI assessment of cartilage defects after 3 months’ healing, the sagittal plane reconstruction of the articular cartilage defect examined, on the left, porcine stifle joint in a 1,2 T high-field MRI setting ((**a**) CAL, (**b**) CAK, (**c**) C); macroscopic and MRI assessment of cartilage defect treated with CAL after 12 months’ healing illustrating the macroscopic picture of the exposed stifle joint after euthanasia (**d**) (sagittal plane reconstruction of the articular cartilage defect examined in a 1,2 T high-field MRI setting and lateral projection, on the left, porcine stifle joint, the medial condyle examined by X-ray in a Philips Digital Diagnost). (Arrows—defect sites.)

The degradation of biomaterials in the defect site, accompanied with the regeneration and formation of the neocartilage and subchondral bone structure, was observed using the MRI and X-ray radiographs after 3 months’ healing. The MRI showed that the neocartilage had a smooth articular surface and that the original articular contour was similar to the adjacent native cartilage; this confirmed that the defect healing of the articular cartilage and the formation of subchondral bone across the whole depth and area of defects with CAL and CAK cements (Figure 8a,b) were complete, contrary to the C sample with incomplete (around 50%) tissue regeneration after 3 months post-surgery. In the defect treated with C cement, the neocartilage was only visible in the defect periphery and a small hole with an unregenerated defect was identified in the center (Figure 8c). The MRI identified a 1.5 mm thickness of neocartilage in the cross-section of the defect in the CAL and CAK samples, but a similar thickness of regenerated cartilage was observed in the defect periphery in the C biocement only. Note that a homogeneous structure of neocartilage with a complete integration of adjacent articular cartilage was revealed in all treated defects. In comparison, the results show that the neocartilage in all treated defects had normal signal intensity, identical to the surrounding cartilage—excluding the central defect region of the C sample. The constitution of the subchondral bone was intact in all defects. The integration of new bone tissue in the CAL and CAK defects into the subchondral bone was almost complete, as compared to the incomplete healing of C. Despite this, remains of not fully identical bone tissue is visible in CAL or CAK cements in some cases after 3 months’ healing. For verification of the tissue stability in regenerated defects, the MRI and X-ray analyses of the tissue after 12-month articular cartilage defect healing treated with the CAL biocement were carried out and they demonstrated successful resorption of CAL cement, as well as an almost full integration without cracks or visible boundaries between adjacent newly formed tissues (hyaline cartilage, subchondral bone) and the original surrounding tissues (Figure 8d). In images, the neocartilage was comparable to the native hyaline cartilage and the morphology of the new bone tissue close to subchondral bone.

## 4. Discussion

Generally, the CPCs were used for the treatment of bone defects [55] and for filling subchondral defects without affecting hyaline cartilage, e.g., in knee, because CPCs were seldom successful for the treatment of damaged cartilage with the formation of morphologically or structurally compatible hyaline cartilage [26,27]. 

Therefore, the CAK and CAL cements represent new relative, simple acellular systems in which an addition of amino acids improves properties that were optimal for osteochondral defect treatment regardless of the shape of the defect site. In CAK and CAL cements, the addition of amino acids caused a strong reduction in CS, from about 40 MPa in C cement to 14 MPa in cements with amino acids. This effect is connected with lower densities of CAK and CAL cements, compared to the C cement, as well as changes in the microstructure and hydroxyapatite particle morphology. While longer needle- or plate-like nanohydroxyapatite particles (up to 200 nm) joined to agglomerates and mutually interconnected via particles on boundaries were observed in C cement, no such morphology or size of nanohydroxyapatite particles was found in amino acid cements. They were instead characterized by very fine globular or thin particles compacted to weaker agglomerates. Moreover, the compactness and strength of HAP particle agglomerates were significantly reduced due to the presence of adsorbed amino acids on the surface of nanoparticles with the same charge, which caused their mutual repulsion and weakening of interconnection. Note that the CS in a wet state did not exceed 5 MPa in both amino acid cements, which significantly improves the mechanical properties of cements in relation to differentiation of bone marrow stem cells to chondrocytes and later formation and growth of cartilage tissue. The insignificant changes in CS of CPC composed of αTCP/DCPD (major content) (around 25 MPa) were only found with LYS increase (up to 2 wt.%) but the addition of LYS caused the rise in both setting time from 27 to 40 min [56]. It was revealed that the nanopillar or nanohole topography and a lower stiffness of film supported the MSC chondrogenesis and facilitated hyaline cartilage, collagen II formation and expression of aggrecan [57].

Although the analysis of the adsorption and release kinetics of used amino acids from cements was not the subject of this paper, we believe that a brief discussion and explanation of the measured dependencies should be made due to their possible use in tissue formation during cell healing processes. In the case of CAK and CAL cements, a large amount (30–50% in dependence on amino acid character) of added amino acids remained bonded to the surface of the created nanohydroxyapatite particles after the transformation of calcium phosphates in cements, which effectively inhibited their crystal growth. It was shown that the amino acids effectively inhibited the hydroxyapatite crystal growth in solution seeded with HAP through adsorption onto the active growth sites of the HAP crystal surface [58,59,60]. Besides the dependence, nanohydroxyapatite particle morphology on the pH environment was revealed during hydroxyapatite precipitation after the addition of ARG; this was due to the change in the ARG amino group charge and the specific adsorption on active sites of hydroxyapatite [61]. The differences in the release rate of individual amino acids between CAK and CAL cements were probably the result of the specific adsorption and charge distribution in ARG and LYS because the composition of cements differs by their contents. It has been confirmed that, despite the small difference between pK_a_ of the α-amino group in LYS and ARG (8.95 and 9.15, respectively), large differences in pK_a_ of guanidinium and terminal amino groups in ARG (13.8) and LYS (10.53) were found [62,63]. Moreover, the positive charge of the guanidinium group is strongly delocalized, which stabilizes the whole structure of the group. These facts significantly influence the behavior of both amino acids, especially in the case of ARG, which indicates the present positive charge in its molecular structure even at very basic conditions, which could locally exist in microporous structures of CAK cement. Measured zeta potentials of stoichiometric hydroxyapatite particles with adsorbed LYS and ARG at pH ˂ 8 were +7.5 and around +30 mV, respectively [61,64], which clearly demonstrates the possible electrostatic interactions between ARG and other negatively charged amino acids in mixtures; this is because their isoelectric points are lower than 7. This effect supported both a much slower release of amino acids from CAK than CAL cement and a low concentration of phosphate ions in solution after soaking (due to mutual electrostatic interactions); furthermore, it probably affected the release of calcium ions from cements. The slower release kinetics of amino acids from the CAK cement caused the presence of thicker amino acid coating on the surface of the cement particles, which hinders diffusion of calcium ions from the surface of the hydroxyapatite particles, despite having a slightly lower stability constant of calcium arginine than calcium lysine complex [65]. The release of LYS from CPC cements was rapid for up to 2 days, from immersion to solution, and finished after 4 days [56]. On the other hand, it was revealed that the adsorption kinetics of LYS are very fast while the release kinetics is slow [66].

For the documentation of osteoblastic activity of cells, the osteogenic gene expressions for osteopontin, osteonectin and collagen I was evaluated by the RT-qPCR analysis. The osteopontin (OP), in a mixture with osteocalcin, promoted hydroxyapatite formation [67]. Osteonectin is a tissue-specific protein that links the bone mineral and collagen phases and is expressed at high levels in osseous tissue with high turnover, such as in active osteoblasts [68,69]. Both proteins and ALP are induced in the mineralization stage of osteoblasts [69] and from this comparison resulted the higher stimulation of osteoblasts to collagen I, OP, ON and ALP expression in CAL than CAK extracts. The ARG actively supported the production and expression of IGF-I by osteoblasts, which stimulate osteoblastic cells to proliferation and differentiation, as well as α1(I) collagen expression and collagen synthesis, proliferation and differentiation of cells with a rise in the ALP activity but reduced the production of osteocalcin (OC) [70,71,72]. The increase in ALP activity of BMSCs, due to their osteogenic differentiation as well as the promoted fibronectin anchoring and the enhanced cellular adhesion, was demonstrated in CPC with the LYS addition [56]. In the case of CAK and CAL cements, the amino acid composition with LYS had a more positive effect on osteogenic activity of osteoblasts than mixture with ARG [73]. Note that LYS plays the crucial role in the cross-linking of the collagen fibers [74].

In vitro testing resulted in the non-cytotoxic character of A and B extracts of all cements. On the other side, the ALP activity of osteoblasts cultured in cement extracts rose with the culture time, but the addition of amino acid to C cement had no stimulating effect on ALP activity.

The CAM assay allows rapid evidence of blood vessel in-growth to the biomaterial before the in vivo testing on animals, and thus evaluates its regeneration capability from the point of view of angiogenesis and biocompatibility. The avian CAM model was previously used as an in ovo method for evaluating the tissue response for the various biomaterials based on collagen, poly-2-hydroxyethyl-methacrylate polymer, polycaprolactone, matrix hydrogels, bioactive glass, polyhydroxybutyrate/chitosan [46,75,76]. Regarding the fact that the CAM assay has been used for several years in the field of regenerative medicine, this type of rapid biocompatibility screening is still an excellent tool to study angiogenic response and speed up the preclinical research process [77]. In the case of the cement extracts in this work, all applied cements induced the angiogenic activity 72 h after implantation with the highest proangiogenic effect of CAL cement, which can support proper bone tissue formation.

The efforts to treat the chondral or subchondral defects without surgery have not successful in articular cartilage regeneration. In principle, the treatment should comprise a minimally invasive procedure and the stimulation of cells to the formation of new tissue with histological and biochemical properties that are similar to those of the native cartilage. On the other hand, migration of the subchondral bone plate can induce the intralesional osteophytes or appearance of subchondral bone cysts in the large osteochondral lesions in patients; this can play a crucial role in the degeneration of repaired cartilage [78]. In a larger study of 180 chondral and osteochondral defect repairs [79], the untreated osteochondral defects showed incomplete filling—mainly with fibrous tissue and small capillary vessels, as well as fibrocartilage in the defect site.

Multiple studies in the past have presented that the repaired cartilage in the articular defect has a different structure to the original hyaline cartilage and inferior mechanical properties [80,81,82]. Contrary to the above facts, the implantation of novel bioactive CAL and CAK biocements into osteochondral defects induced regeneration of the subchondral bone, as well as of hyaline cartilage. Macroscopic analysis demonstrated the formation of smooth cartilaginous white tissue with a slightly irregular surface in defects treated with CAL and CAK cements. More detailed analysis showed the formation of hyaline cartilage and new subchondral bone without reduction in thickness, alterations in architecture and cluster formation, as well as almost natural interconnection between new subchondral bone and neocartilage. In many studies, interconnection of neocartilage with surrounding native cartilage has seldom been confirmed [83,84,85]. The margins of the neocartilage were well joined with the adjacent tissue in CAL and CAK samples, with no cracks in integration zones, whereas integration with surrounding cartilage was incomplete in C sample. Thus, it is clear that the amount of amino acids and the composition of their mixture in the cements (regardless of whether LYS or ARG was in the mixtures) were appropriate and significantly improved the formation of proper architecture and morphology of neocartilage. It is probable that amino acid mixtures in cements supported differentiation of the MSCs to chondrocytes and facilitated biosynthesis of collagen due to the enlarged content of basic amino acid components without the need for additional endogenous synthesis in cells.

Cell density and chondrocyte morphology in all layers, as well as zone structure, correspond to native hyaline cartilage. The MRI evaluation showed normal signal intensity, identical to adjacent cartilage and homogeneous structure of the neocartilage. It is clear from the comparison of tissue morphology that the new tissue in defects filled with C cement was different to the others, from a macroscopic and histological point of view. The defect was covered with translucent cartilaginous tissue and was concavely depressed with a small amount of fluid. In addition, the boundary between the adjacent and normal cartilage was more visible and the integration with origin tissue was incomplete. Hypocellularity was observed in the central part of the neocartilage, as compared with the CAL- and CAK-treated defects. Note that the histological observations revealed no increase in the inflammatory cell number and biocements did not cause any inflammatory reaction. No fibrous tissue was observed in the regenerated cartilage, indicating the effective support of applied biocements for differentiation of MSCs to required cell lines after their infiltration to the defect site. The subchondral bone had been completely repaired, and there was no difference compared to the repaired site with the adjacent subchondral bone tissue.

Compared to other techniques of the osteochondral tissue engineering [27], the surface incongruency of the treated defect with adjacent cartilage could be eliminated by using CPC biocements, which can be applied to defects of various sizes and shapes in paste form after self-setting. Therefore, it is possible to reflect the natural curvature of the femoral condyle and take into account the individual differences in size of defects by applying cements. Moreover, fast setting and resistance to wash-out of CAK and CAL cements, after being introduced to the defect site, are the positive characteristics for preventing early-stage implant failure or dispersion of the solid biomaterial after immediate contact with physiological fluids or when bleeding occurs. From the point of view of the applied amino acids, the high addition of glycine, as well as lower supplementation of culture media with LYS or PRO in comparison with GLY, induced strong in vitro production of collagen II by chondrocytes [31]. Despite this, a direct effect of ARG on in vitro collagen synthesis has not been shown; ARG significantly supports wound healing and stimulates in vitro and in vivo T-cell responses, as well as reduces the negative effect of injury on T-cell function [86], which likely had a positive influence in the osteochondral defect healing in the case of CAK cement. It is known that the addition of PRO to medium or the rise in its extracellular concentration had a small, but significant, effect on in vitro collagen biosynthesis by fibroblasts. On the other hand, it was found that there was a strong positive effect of extracellular PRO on collagen biosynthesis when insufficient endogenous synthesis of PRO occurred from substrates, such as glutamine [35].

The applied surgery procedure based on deeper drilling to subchondral bone (to a depth of 1 cm), in combination with a pasty consistency of cements, allows massive bone-marrow flow with a complex composition of cells, growth factors and cytokines, as well as mesenchymal stem-cell migration from bone cavity to artificial defect. Treatment of the defect site by this procedure eliminated the utilization of additional invasive procedures connected with autologous harvesting of MSCs or chondrocytes from patients and their long-term cultivation in the next step. The cellular composition of the metaphyseal compartment and cell activity differs by location and distance from bone surface, which can significantly support remodeling and growth of new bone tissue due to enhanced activity of the bone cells [87]. Platelet-rich plasma and bone marrow concentrate are known to have a positive effect on the formation and long-term stability of neo-cartilage and improve cartilage repair, as compared to microfracture [88]. Note that applied biocements were almost fully resorbed during healing.

## 5. Conclusions

The novel tetracalcium phosphate/monetite biocements with a 4 wt.% addition of amino acid mixture, characteristic of collagen (GLY, PRO, HYP and LYS or ARG), were characterized in terms of their material properties; furthermore, they were in vitro tested and successfully in vivo applied for treatment of osteochondral defects in large animal model (pigs). Synthesized cements with a simple composition were used; they were prepared in acellular form after mixing with hardening liquid and, then, in paste form they were introduced to the knees of animals after drilling the artificial osteochondral defects. The amino acids and calcium ions were rapidly released form cements during the first stage of the setting process, whereas 30–60% amino acids, dependent on the type of amino acid, remains adsorbed on hydroxyapatite particles in cements. It was revealed that there was about a 60% reduction in the compressive strength (especially in the wet state), as well as particle refinement in cements after the addition of the amino acid mixture. The measured material characteristics of biocements likely supported, positively, the proper formation of hyaline cartilage and subchondral bone defect healing. The RT-qPCR gene expression confirmed in vitro over expression of osteogenic genes in osteoblasts after 7 days of culture in CAL extracts, and no cytotoxicity of cement extract was identified. Results from macroscopic, histological and radiological analyses clearly showed the excellent healing process of artificial osteochondral defects in pigs after treatment with CAL and CAK cements without any inflammation, as well as formation of subchondral bone and hyaline cartilage that were morphologically and structurally identical with the original tissues. Good integration of hyaline neocartilage with the surrounding tissue, as well as perfect interconnection between neocartilage and new subchondral bone tissue, was demonstrated.

## Figures and Tables

**Table 1:** Forward and reverse primers of genes used for RT-qPCR experiments.

Gene	Primers (5′–3′)	Product Length (bp)	Reference
β-actin mouse	F: CTCTGGCTCCTAGCACCATGAAGA	200	[44]
	R:GTAAAACGCAGCTCAGTAACAGTCCG		
Type I collagen mouse	F: CTCCTGACGCATGGCCAAGAA	100	[45]
	R: TCAAGCATACCTCGGGTTTCCA		
Osteopontin mouse	F: TGATTCTGGCAGCTCAGAGGA	110	[45]
	R: CATTCTGTGGCGCAAGGAGATT		
Osteonectin mouse	F: ATGTCCTGGTCACCTTGTACGA	103	[45]
	R: TCCAGGCGCTTCTCATTCTCAT		
Alkaline phosphatase mouse	F: ACCCGGCTGGAGATGGACAAAT	113	[45]
	R: TTCACGCCACACAAGTAGGCA		

## Data Availability

Data is contained within the article.
